# Visual Prognosis after Explantation of a Corneal Shape-Changing Hydrogel Inlay in Presbyopic Eyes

**Published:** 2019

**Authors:** Majid Moshirfar, Benjamin Buckner, David B. Rosen, Madeline B. Heiland, Yasmyne C. Ronquillo, David F. Skanchy, Harry Y. Liu, Tim Melton, Liliana Werner, Phillip C. Jr Hoopes

**Affiliations:** 1 John A. Moran Eye Center, Department of Ophthalmology and Visual Sciences, School of Medicine, University of Utah, Salt Lake City, UT, USA; 2 Utah Lions Eye Bank, Murray, UT, USA; 3 Hoopes Durrie Rivera Research Center, Hoopes Vision, Draper, UT, USA; 4 College of Medicine-Phoenix, University of Arizona, Phoenix, AZ, USA; 5 Department of Ophthalmology and Visual Sciences, W.K. Kellogg Eye Center, University of Michigan Medical School, Ann Arbor, Michigan, USA; 6 Health Science Center, McGovern Medical School, University of Texas, Houston, TX, USA

**Keywords:** Raindrop Corneal Inlay, Explantation, Presbyopia, Visual Prognosis, Corrected Distance Visual Acuity

## Abstract

The purpose of this case series is to report visual outcomes in patients who underwent explantation of the Raindrop® hydrogel corneal inlay. Retrospective chart review comprising four cases of explantation of the Raindrop® corneal shape-changing hydrogel inlay: pre-implantation, pre-explantation, and post-explantation values for uncorrected distance visual acuity, uncorrected near visual acuity, and corrected distance visual acuity (CDVA) were measured; keratometric and tomographic data were collected using the Pentacam system (Oculus, Inc). Three eyes were explanted for progressive haze after implantation that persisted even after removal; one eye was explanted due to poor visual acuity with no haze formation. All patients experienced decreased unaided and corrected distance visual acuity. Persistent increase in corneal thickness and mean keratometry was noted post-explantation. All four patients regained their original near visual acuities, but one patient had persistent one-line loss in CDVA. There are long lasting tomographic corneal changes following Raindrop inlay explantation. In addition, persistent increased corneal thickness could be related to semi-permanent changes in corneal structure and may account for residual haze experienced by patients. After explantation, patients may not return to baseline CDVA.

## INTRODUCTION

**Figure 1:**
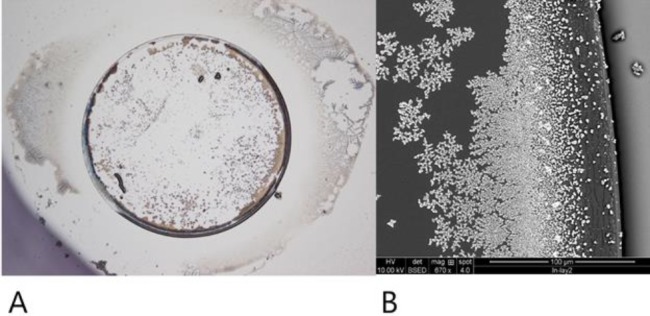
A: Light microscopy of explanted Raindrop^®^ inlay, explanted from case 1 at 20x magnification. The specimen appears to be quite intact. Note that the deposits appear to be mostly related to the balanced salt solution as they are also observed on the glass slide outside of the area of the device. B: Scanning electron microscopy photograph of the inlay, showing the fern-like deposits on its surface

The Raindrop® Near Vision Inlay (ReVision, Inc., Denver, CO, USA) received US FDA approval for use in the United States on June 29, 2016 after being determined to have reasonable safety and efficacy [1]. The Raindrop® inlay is a transparent microscopic hydrogel-based corneal inlay (2millimeter [mm] diameter, 32micrometer [μm] thick, placed between 100-200μm depth) [2] designed to create a prolate-shaped cornea to correct near visual acuity in the non-dominant eye (Fig. 1). The insertion of the inlay into the central corneal stromal bed increases stromal volume, which presses against the anterior cornea leading to an increase in central corneal curvature. This curvature change increases the refractive power of the central cornea by a few diopters, providing enhanced near visual acuity in presbyopic patients with the goal of improved distance and near visual acuity relying on the principle of monovision [2]. This product is no longer distributed in the United States after an FDA safety warning issued in 2018 due to corneal haze [3, 4]. 

One study found that adverse events generally took place in one of two periods, with the first occurring in the first 3 months postoperatively. Common adverse reports during this period were flap-related issues, pressure spikes, and inlay exchanges. The second period, from 3 months through 1 year, concerned mainly issues with inlay explants and transitory loss of corrected distance visual acuity; a small percentage of patients were reported to have central stromal haze during this period [5]. In addition, the more recent FDA report suggests that haze occurring after one year is a significant reason for explantation [4]. The purpose of this study is to highlight outcomes of patients who underwent Raindrop explantation. Data presented as mean, frequency, and percentage.

## METHODS

This is a retrospective case series of patients who underwent Raindrop inlay implantation at our facility between December 2016 and April 2017. Inclusion criteria for implantation were: age 41-65, manifest refractive spherical equivalent + 1.00 to -0.5 diopters (D) and ≤ 0.75 D cylindrical refraction, requiring + 1.50 to + 2.50 D for near vision [6]. Charts of patients who underwent explantation between December 2017 and April 2019 were reviewed. Information was collected on pre-implantation, pre-explantation, and post-explantation values for uncorrected near visual acuity (UNVA), uncorrected distance visual acuity (UDVA), and best corrected distance visual acuity (CDVA). Keratometric data on central corneal thickness (CCT) and mean keratometry (Km) was obtained from the Pentacam system (Oculus, Inc. Arlington, WA, USA). Approval was obtained from the Hoopes Research Committee, and informed consent was signed by each patient. All procedures adhered to the tenets of the Declaration of Helsinki.

## RESULTS

Eight patients had Raindrop implantation during the study period. Four patients (50%) had subsequent explantation. The mean duration of implantation was 1.5 years (range 1 to 2.5 years) with a mean flap thickness of 175 μm (range 170-180 μm). The most recent data available was, on average, 242 days post-explantation (range 8-356 days). All four patients complained of blurry vision. Two patients complained of discomfort while the inlay was in place; both reported resolution of discomfort within weeks of explantation. Three patients complained of “seeing shadows” while the inlay was in place.

**Figure 2:**
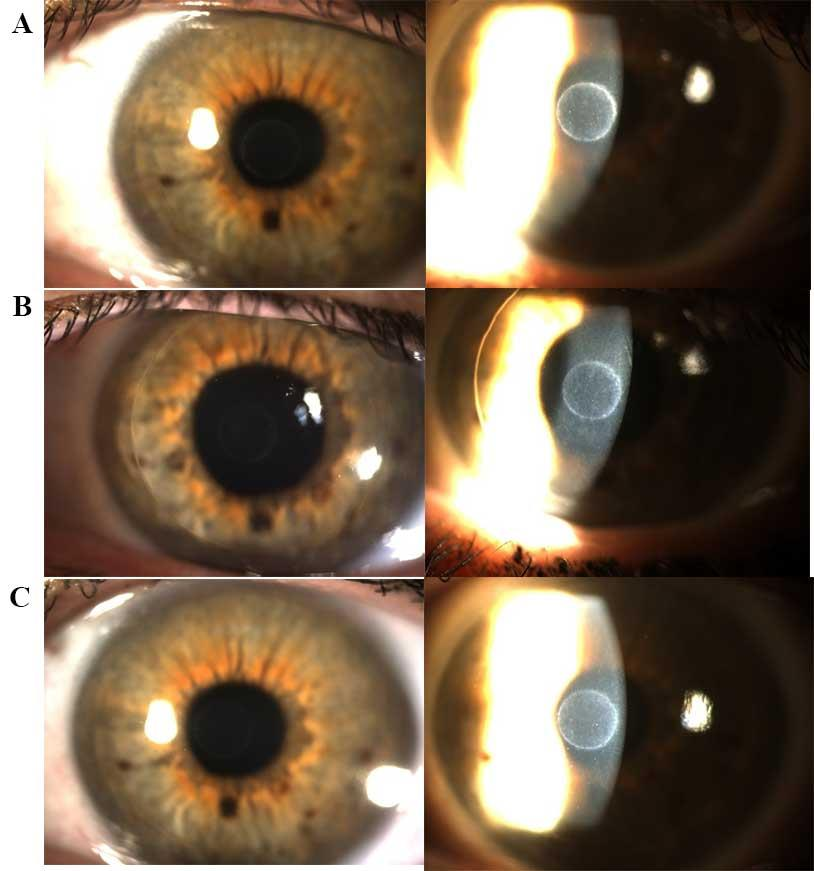
Photographs of case 1 taken immediately before (A), 1 day after (B), and 1 month after (C) explantation of the Raindrop^® ^corneal inlay. Note that the haze appears to be subsiding slightly in the more direct lighting conditions but lingers with the tangential illumination. The observer may mistakenly believe the inlay is still in place when it is in fact explanted and there is only residual haze and fibrosis

Three patients had stromal haze that developed during implantation and persisted after explantation (Fig. 2). The haze resolved at the 1-year post-explantation follow up in two of the patients (duration of inlay implantation 16 and 17 months, respectively). The third patient with persistent haze has not completed a 1-month post-explantation exam (inlay was in place for 28 months). Out of the four eyes, only one patient did not experience haze during implantation or after removal; this patient’s inlay was in place for 12 months.

All patients regained or improved in UNVA compared to the pre-implantation value (mean increase 1.5 lines, Snellen) (Table 1). Uncorrected distance visual acuity changes ranged from 0-6 lines lost (mean loss of 4 lines, Snellen). Data on CDVA post-explantation was available in three out of four patients; one patient had persistent one-line loss of CDVA on last follow up (Table 1). No hyperopic shifts were recorded at any point during implantation. Mean keratometry had a mean increase of + 2.5 D after implantation (Table 2). Manifest refraction spherical equivalent (MRSE) on all four patients showed a myopic shift before removal with mean decrease of -0.75 D; there was a persistent myopic shift compared to baseline in all three patients that had post-explantation MRSE (mean -0.46 D) (Table 3).

**Figure 3:**
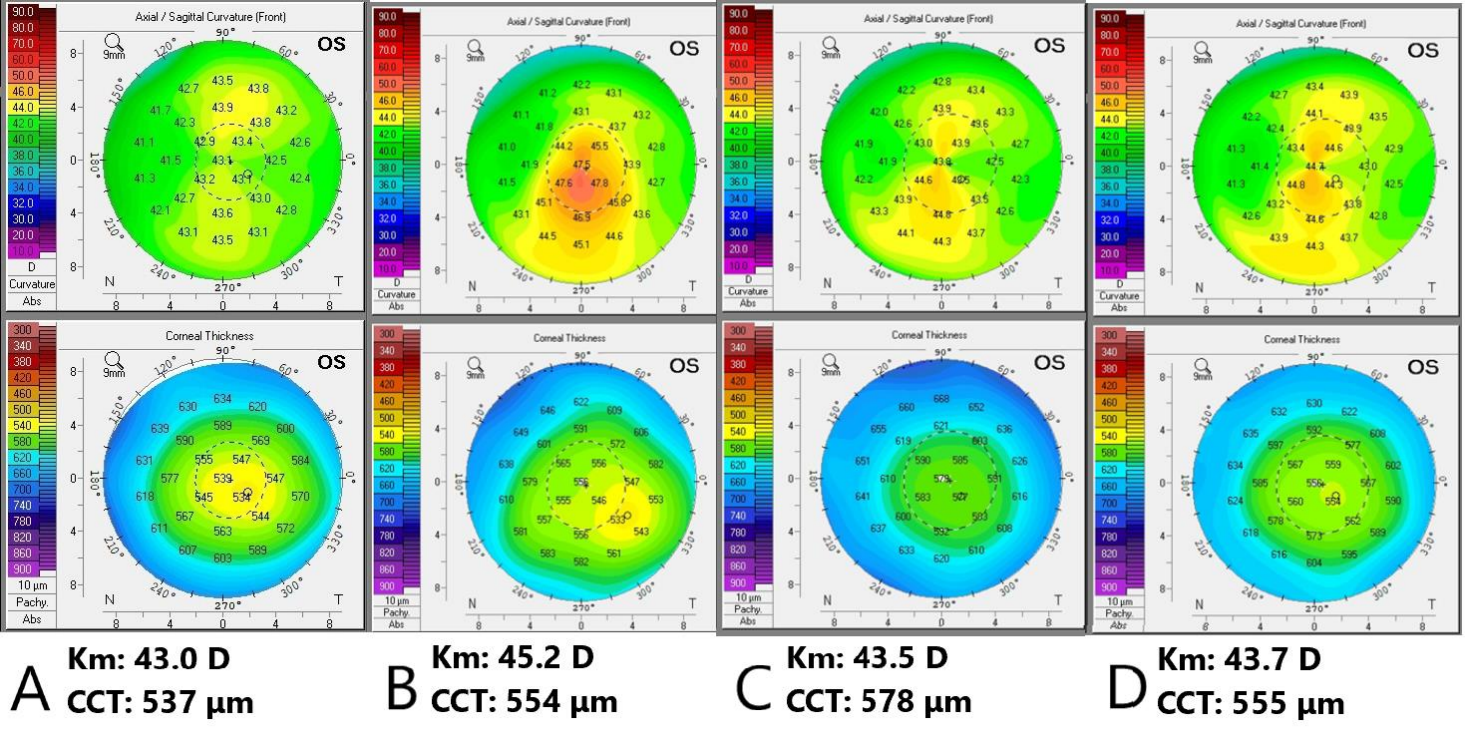
PENTACAM data for case 1 of the Raindrop^®^ Hydrogel Corneal Inlay

Central corneal thickness had a mean increase of 27 μm after implantation. Keratometric and tomographic data was available post-explantation in two patients with a Km increase of + 0.7 D and an increased CCT of 10-16 μm compared to pre-implantation (Table 2; Fig. 3).

**Figure 4:**
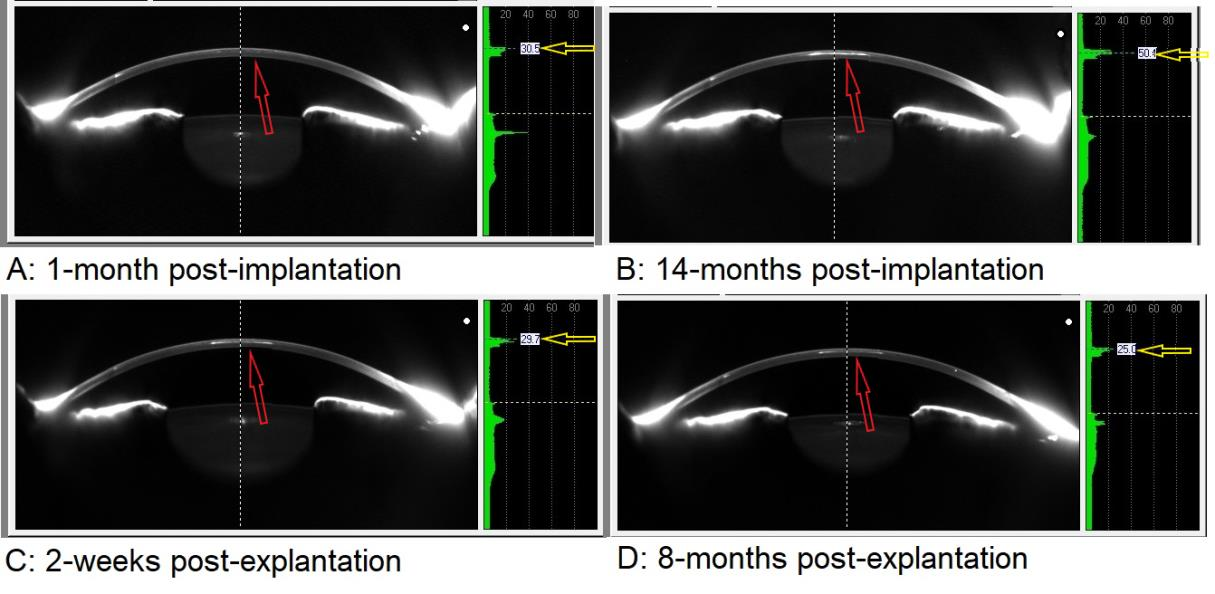
Densitometry from PENTACAM for case 1 of the Raindrop^®^ Hydrogel Corneal Inlay at various time-points

Densitometry was done using the Pentacam system on patient 1 and showed an increase of 19.9 standard gray scale units (GSU) over 13 months of implantation. The density decreased by 25.0 GSU 8 months after removal (Fig. 4). Upon explantation, the inlay was placed on a glass slide and allowed to dry at room temperature. It was then forwarded to the Intermountain Ocular Research Center (University of Utah) for laboratorial analysis. Evaluation under a light microscope (Olympus Optical Co., Ltd.) (Fig. 1) showed the presence of deposits on the inlay, with a fern-like morphology that appeared to correspond to dried salts.

The specimen (inlay on glass slide) was then coated with gold-palladium, and then analyzed under scanning electron microscopy (SEM - Quanta 600F, ThermoFisher Scientific), coupled with energy dispersive spectroscopy (EDS) for elemental analysis of the deposits. The analyses were done under high vacuum pressure (approximately 10-6 torr), at room temperature, and an accelerating voltage of approximately 10-15 kV. SEM/EDS confirmed that the deposits on the inlay were mostly composed of sodium (Na) and chlorine (Cl).

**Table 1:** Changes in Visual Acuity Pre- and Post-explantation of the Raindrop® Hydrogel Corneal Inlay

	UDVA			UNVA			CDVA		
Patients	**Pre-inlay**	**Pre-explant**	**Post-explant**	**Pre-inlay**	**Pre-explant**	**Post-explant**	**Pre-inlay**	**Pre-explant**	**Post-explant**
1	20/25	20/300	20/80	20/100	20/40	20/63	20/20	20/40+2	20/25+
2	20/20	20/25	20/20-	20/50	20/40	20/50	20/20	20/25+2	20/20-
3	20/20	20/70-2	20/40+2	20/100	20/50	20/100	20/20	20/25-	20/20
4	20/20-2	20/50	20/50	20/100	20/80	20/100	20/20	20/40	NA

Abbreviations: CDVA: corrected distance visual acuity; UDVA: uncorrected distance visual acuity; UNVA: uncorrected near visual acuity; NA: not available; Pre-inlay: before implantation of inlay; Pre-explant: before explantation of inlay; Post-explant: after explantation of inlay

**Table 2:** Changes in keratometry and CCT Pre- and Post-explantation of the Raindrop® Hydrogel Corneal Inlay

	Pre-Implantation		Pre-explantation		Post-explantation	
Patients	**Km(D)**	**CCT (μm)**	**Km(D)**	**CCT (μm)**	**Km(D)**	**CCT (μm)**
1	43	537	45.2	554	43.7	555
2	45	554	45.2	565	NA	NA
3	43.2	580	48.8	632	43.9	596
4	42.4	559	44.4	586	NA	NA

Abbreviations: Km: mean keratometry; CCT: central corneal thickness; D: diopter; μm: micrometer; NA: not available

**Table 3:** Changes in manifest refraction Pre- and Post-explantation of the Raindrop® Hydrogel Corneal Inlay

Patient	MRSE (D) Pre-implantation	MRSE (D) Pre-explantation	MRSE (D) post-explantation	Change from baseline
1	-0.375	-1.75	-1.375	-1
2	-0.125	-0.25	-0.25	-0.125
3	+0.75	-0.125	+0.5	-0.25
4	+0.5	-0.125	NA	NA

Abbreviations: MRSE: manifest refraction spherical equivalent; D: diopter; NA: not-available.

**A) shows pre-implantation data, with a Km of 43.0 D and CCT of 537 μm. B) shows pre-explantation data with a Km of 45.2 D and CCT of 554 μm. C) shows 1-day post-explantation data with a Km of 43.5 and CCT of 578 μm. D) shows 1-month post-explantation data with a Km of 43.7 and CCT of 555 μm.**

**Abbreviations: Km: mean keratometry; D: diopter; CCT: Central corneal thickness; μm: micrometer.**

**Red arrows point to corneal stromal haze and yellow arrows point to the corresponding densitometry measurement. Densitometry values were as follows: 30.5 grayscale units (GSU) at 1-month post-implantation; 50.4 GSU at 14-months post-implantation; 29.7 GSU at 2-weeks post-explantation; and 25.0 GSU at 8-months post-explantation. Density increases with the duration of implantation. After explantation, density decreases with time. We infer that the increase in density correlates with corneal haze.**

## DISCUSSION

The results of this case series highlight reasons for the high explantation rate of the Raindrop and visual prognosis after removal. There appeared to be no changes to the inlay over the course of implantation. Haze was a very common problem and was persistent up to 1 year after explantation. Tomography and keratometry demonstrated corneal shape changes induced by the inlay; these changes persisted to some degree after explantation.

Garza et al. reported a case of explantation due to unsatisfactory visual outcomes [7]. Chayet et al. reported another case of explantation that occurred in a patient that had LASIK post-inlay implantation and experienced haze [8]. In the FDA post-approval study for Raindrop, there is a 23% explant rate over 5 years [4].

Normal density values (for eyes without history of corneal inlay) based on densitometry measurements are reported to be around 20 GSU [9]. Our data showed an increase in density with duration of implantation (Fig. 4). We postulate that corneal haze and increased density may be secondary to inflammatory changes induced by the implant. Both haze and density decreased over months after removal. Resolution of haze may occur through the cornea’s restorative mechanisms. This includes epithelial healing via limbal stem cells, basement membrane remodeling, and stromal healing via keratocyte motility and Transforming growth factor beta (TGF-beta) activation [10]. Densitometry has been used to monitor haze in other patients with a corneal inlay [11]. We suggest that densitometry is a more objective way to measure haze-related changes induced by corneal inlays. The long-term recoverability of these impacted corneas remains to be seen, and additional studies should be conducted to investigate this phenomenon.

Corneal tomography showed an expected Km increase after implantation. However, Km and CCT were persistently elevated even after explantation, demonstrating that there were residual changes in corneal shape. This could explain why two of the patients still maintained better UNVA even after inlay removal. Despite these changes, all tomographic values trended toward baseline on latest follow-up (Table 2; Fig. 3). Longer follow up is needed to determine if corneal shape will return to baseline after inlay removal.

As a point of comparison, the KAMRA® inlay also has studies on its explantation [12, 13]. A recent study of KAMRA® explantation from Shing Ong et al. found that one major complication during implantation was corneal haze and accompanying hyperopic refractive shifts [12]. It was also found that after explantation the vision trended back toward baseline values and the authors concluded that early removal led to better visual outcomes [12]. Haze was a similar reason for explantation of the Raindrop, although hyperopic shifts were not reported in our series. Both inlays trend toward baseline values after removal. However, the method of insertion – flap insertion (Raindrop®) and pocket insertion (KAMRA®), the insertion depth, and the mechanism of action differ significantly between the two inlays.

There were several limitations to this study. First, there was a very small sample size. The study was retrospective in nature and limited by the amount of information available in the chart. Follow up times varied widely, with minimal post-explantation data available on one patient. Perhaps a larger sample size with more consistent follow up could bring greater insight to factors associated with development and resolution of haze. If more patients had data available on tomography and densitometry, more robust conclusions could be made as to the changes induced by the Raindrop corneal inlay.

## CONCLUSION

Despite the small sample size and retrospective nature of the study, we conclude that the Raindrop is associated with significant adverse effects, the most common being blurry vision and stromal haze. Because of the small sample size, it is difficult to establish a temporal relationship between haze development and resolution. Persistent shape changes may account for improvement of UNVA after removal, but deficits in UDVA are common with potential for persistent loss of CDVA. In addition, tomographic and density changes persisted post-explantation (Figs. 3 and 4). Patients should not expect to return to pre-implantation values in the short term after removal. As per FDA advice, patients with Raindrop implants should follow-up regularly, even after explantation, due to the development of corneal haze [4]. Alternative synthetic inlays other than the Raindrop® exist [7, 14-20]. However, allogenic implants such as the presbyopic allogenic refractive lenticule (PEARL) [21] and TransForm corneal allograft (TCA) [22] are undergoing clinical trials. Perhaps using an allogenic implant will result in a lower complication rate and will not induce as visually-disturbing corneal haze as synthetic inlays such as the Raindrop.

## DISCLOSURE

Ethical issues have been completely observed by the authors. All named authors meet the International Committee of Medical Journal Editors (ICMJE) criteria for authorship of this manuscript, take responsibility for the integrity of the work as a whole, and have given final approval for the version to be published. No conflict of interest has been presented. Phillip C Hoopes Jr, MD is a consultant for CorneaGen.

## Funding/Support:

Research to Prevent Blindness, NY, USA
